# Impact of Elevated Blood Pressure Targets on Splanchnic Ischemia and Mortality in Septic Resuscitation: Systematic Review

**DOI:** 10.2196/98772

**Published:** 2026-10-27

**Authors:** Paulina Cisneros, John Fredy Ortiz Caviedes, Lina Maria Piñeros Ladino, Natalia Quintero, Valeria García Oñate Cervera, Juan David Urrea Ospina, Erika Patricia Cerna Alcantar

**Affiliations:** 1 Cardiology Department, Guthrie Forssmann Cardiovascular Institute, Hospital Enrique Garcés, Chilibulo S/N y Enrique Garcés, Quito, Ecuador, 593 0999009697; 2 Internal Medicine Department, Hospital San Rafael, Bogota, Colombia; 3 Medicine Department, Preventivamente Salud IPS, Medellín, Colombia; 4 Clinical Toxicology Department, Fundación Universitaria de Ciencias de la Salud FUCS, Bogotá, Colombia; 5 Medical Department, Escuela Medico Militar, Ciudad de México, Mexico; 6 Medical Department, Universidad Libre de Colombia, Cali, Colombia; 7 Internal Medicine Department, Mexican Social Security Institute, San Luis Potosí, Mexico

**Keywords:** septic shock, mean arterial pressure, vasopressor therapy, hemodynamic resuscitation, splanchnic perfusion, acute kidney injury

## Abstract

**Background:**

Sepsis causes deaths worldwide each day, with hemodynamic resuscitation being central to management. Guidelines recommend a 65-mm Hg mean arterial pressure (MAP) target, yet the evidence for elevated targets remains fragmented across mortality, renal, and splanchnic outcomes.

**Objective:**

This review aims to comprehensively appraise the impact of elevated MAP targets on splanchnic ischemia, mortality, and organ-specific outcomes in septic resuscitation.

**Methods:**

Following the PRISMA (Preferred Reporting Items for Systematic Reviews and Meta-Analyses) 2020 guidelines, we systematically searched PubMed, the Cochrane Library, and Google Scholar for studies comparing MAP targets in septic shock. Risk of bias was assessed using the Cochrane Risk of Bias 2 (RoB 2; randomized controlled trials [RCTs]) and the Newcastle-Ottawa Scale (nonrandomized studies). The findings were synthesized narratively according to the Synthesis Without Meta-Analysis guidelines, with random-effects meta-analysis for mortality outcomes. Evidence certainty was graded using the Grading of Recommendations Assessment, Development, and Evaluation (GRADE) framework.

**Results:**

From 3254 records, 11 studies (2895 patients; 4 RCTs, 7 nonrandomized) met the inclusion criteria. Pooled 90-day mortality across 3 trials showed a nonsignificant trend toward higher mortality with elevated MAP targets (320/720, 44.4% vs 280/722, 38.8%; OR 1.31, 95% CI 0.97‐1.77). Pooled 28- or 30-day mortality showed a similar nonsignificant trend (OR 1.25, 95% CI 0.98‐1.61), which was robust to sensitivity analysis. Narratively, mortality findings across nonpooled observational studies were inconsistent; some linked low MAP to higher death risk, and others found no significant association. Renal outcomes were inconsistent, with some studies showing reduced renal replacement therapy or improved acute kidney injury recovery at higher targets, particularly in hypertensive or cirrhotic subgroups. Elevated MAP targets consistently increased vasopressor exposure and were associated with more arrhythmias and fewer organ support–free days. No study reported splanchnic ischemia as a discrete outcome; mesenteric ischemia rates were low and similar between arms. Overall certainty of evidence was very low.

**Conclusions:**

Higher MAP targets show no clear mortality or splanchnic perfusion benefit and may increase overall harm, though select subgroups, chronic hypertension, and cirrhosis, may benefit, supporting individualized, risk-stratified MAP targeting over uniformly elevated-target strategies.

## Introduction

Sepsis affected an estimated 48.9 million people and caused 11 million deaths worldwide in 2017 (nearly 1 in 5 deaths globally), equating to more than 30,000 deaths per day [1]. Globally, sepsis incidence rose by 230% among individuals aged 15 years and older between 1990 and 2021, with a further 30% surge during 2020‐2021 alone amid the COVID-19 pandemic, underscoring an urgent, sustained need for advances in sepsis therapy [2]. Due to socioeconomic difficulties and a lack of thorough epidemiological surveys, sepsis mortality continues to exist in low-income countries [3].

Maintaining a mean arterial pressure (MAP) of 65 mm Hg is advised by current sepsis management recommendations, which state, as a strong recommendation based on moderate-quality evidence, that there is no appreciable additional benefit to targeting a higher MAP in patients with septic shock [4]. An MAP of more than 70 mm Hg has been shown in several trials to improve microcirculation during septic shock and to avoid acute renal damage [5,6]. In patients with sepsis, hemodynamic control is an essential part of the overall therapeutic strategy [7], as it plays a critical role in early vital sign stabilization, anticipating disease progression, and avoiding further tissue and organ failure [8]. However, maintaining a high MAP may need higher doses of vasoactive medications, which might result in further issues and bodily harm [9].

Despite guideline endorsement of a 65-mm Hg MAP target in septic shock, the evidence base for elevated MAP targets remains fragmented and outdated. Leone et al [8] synthesized only studies through 2014, predating 3 pivotal trials, with the latter halted early for harm, leaving current practice uninformed by the most recent and arguably most consequential data. Dari et al [10], while more recent, pooled only 4 randomized controlled trials (RCTs) and excluded observational and cohort evidence entirely, yielding a narrower and potentially incomplete effect estimate. A study by Kattan et al [11] offered valuable mechanistic and monitoring insight but remained a narrative review, lacking quantitative synthesis or a dedicated, pooled assessment of splanchnic and mesenteric outcomes. Critically, no prior review has directly synthesized splanchnic or mesenteric ischemia as a discrete, cross-study outcome nor systematically reconciled short- and long-term mortality signals alongside renal, hemodynamic, and organ-support end points across both randomized and observational designs. Our review addresses these gaps by incorporating the most contemporary RCTs and observational cohorts, applying sensitivity analyses to resolve heterogeneity, and explicitly evaluating splanchnic perfusion alongside mortality. The aim of this review is to comprehensively and quantitatively appraise the impact of elevated MAP targets on splanchnic ischemia, mortality, and organ-specific outcomes in septic resuscitation.

## Methods

### Study Design

This systematic review was conducted and reported in accordance with the PRISMA (Preferred Reporting Items for Systematic Reviews and Meta-Analyses) 2020 guidelines and checklist (Checklist 1) [12]. The review protocol was developed a priori, with the research question, eligibility criteria, search strategy, and outcome hierarchy defined before data extraction commenced. The review was not registered prospectively on PROSPERO, which is acknowledged as a limitation.

### Eligibility Criteria

#### Inclusion Criteria

The inclusion criteria were as follows:

- Adult patients (≥18 y) diagnosed with sepsis, severe sepsis, or septic shock according to Sepsis-2 or Sepsis-3 consensus definitions.
- Patients admitted to the intensive care unit (ICU) requiring vasopressor-supported hemodynamic resuscitation.
- Studies involving septic shock in patients with underlying comorbidities (eg, cirrhosis, chronic kidney disease, or chronic hypertension) were eligible provided that MAP-related clinical outcomes were reported separately.
- RCTs, quasi-randomized trials, prospective interventional studies, post hoc analyses of clinical trials, and prospective or retrospective observational cohort studies.
- Studies evaluating different MAP targets or achieved MAP levels during septic shock resuscitation, including higher MAP targets (generally ≥75‐85 mm Hg), standard MAP targets (generally 60‐70 mm Hg), or analyses of MAP as a continuous or categorical exposure variable.
- Studies reporting at least one of the following outcomes:
  - Primary outcomes: all-cause mortality or splanchnic perfusion- or ischemia-related outcomes.
  - Secondary outcomes: acute kidney injury (AKI), renal replacement therapy (RRT), organ dysfunction (eg, Sequential Organ Failure Assessment [SOFA] score), lactate, hemodynamic parameters, vasopressor requirements, mechanical ventilation, ICU or hospital length of stay, or adverse events.

#### Exclusion Criteria

The exclusion criteria were as follows:

- Studies involving nonseptic populations, or mixed populations in which data for patients with septic shock could not be extracted separately.
- Pediatric populations (<18 y).
- Studies evaluating vasoactive therapy without reporting MAP targets or achieved MAP levels.
- Case reports, case series with fewer than 10 patients, editorials, letters, narrative reviews, conference abstracts without full text, protocols, animal studies, and in vitro studies.
- Studies not reporting any predefined primary or secondary clinical outcome relevant to this review.
- Duplicate publications or secondary analyses of the same dataset, unless they reported additional relevant outcomes not available in the primary publication.

### Information Sources and Search Strategy

A comprehensive systematic literature search was conducted across 3 electronic bibliographic databases: MEDLINE (via PubMed), Google Scholar, and the Cochrane Library. No restrictions were placed on publication date or language during the initial search. However, due to resource limitations, only studies with full-text articles available in English were included in the final review, which may have introduced language bias.

The search strategy was developed using a combination of MeSH and free-text keywords based on the Population, Intervention, Comparator, and Outcome (PICO) framework. For PubMed, the following search strategy was applied: (“Sepsis”[Mesh] OR “Shock, Septic”[Mesh] OR sepsis OR “septic shock” OR “severe sepsis”) AND (“Blood Pressure”[Mesh] OR “Mean Arterial Pressure” OR MAP OR “mean arterial pressure” OR “blood pressure target” OR “higher MAP” OR “high MAP” OR “elevated blood pressure” OR “hemodynamic resuscitation” *OR vasopressor* OR norepinephrine OR vasopressin) AND (“Splanchnic Circulation”[Mesh] OR “Mesenteric Ischemia”[Mesh] OR splanchnic OR “splanchnic perfusion” OR “splanchnic ischemia” OR mesenteric OR gut OR intestinal OR gastrointestinal OR gastric OR hepatic OR liver OR renal) AND (“Mortality”[Mesh] OR mortality OR death OR survival OR “Organ Dysfunction Scores”[Mesh] OR “organ dysfunction” OR “multiple organ failure” OR “acute kidney injury” OR AKI OR lactate).

For the Cochrane Central Register of Controlled Trials (CENTRAL), an adapted search strategy using free-text terms was applied: (sepsis OR “septic shock” OR “severe sepsis”) AND (“mean arterial pressure” OR MAP OR “blood pressure target” OR “higher MAP” OR “high MAP” OR “elevated blood pressure” OR “hemodynamic resuscitation” OR vasopressor OR norepinephrine OR vasopressin) AND (splanchnic OR “splanchnic perfusion” OR “splanchnic ischemia” OR mesenteric OR intestinal OR gastrointestinal OR gastric OR hepatic OR liver OR renal) AND (mortality OR death OR survival OR “organ dysfunction” OR “multiple organ failure” OR “acute kidney injury” OR AKI OR lactate).

Database-specific syntax and Boolean operators were adapted as appropriate for each platform. For Google Scholar, a simplified combination of key search terms based on the PICO framework was used, and the first 400 results, sorted by relevance, were screened because of the platform’s limitation in exporting large numbers of citations. In addition, the reference lists of all included studies and relevant review articles were manually searched to identify any additional eligible studies that were not retrieved through the electronic database searches.

### Study Selection

#### Overview

Search results from all databases were collated and deduplicated using reference management software. Two independent reviewers screened titles and abstracts against the prespecified eligibility criteria. Studies judged potentially eligible at the abstract screening stage were retrieved as full-text articles and subjected to detailed eligibility assessment. Disagreements between reviewers at any stage of screening were resolved through discussion and, where necessary, by referral to a third reviewer. The reasons for exclusion at the full-text stage were documented and are summarized in the PRISMA flow diagram. Pettilä [13] et al (TARTARE-2S, 2026) was excluded as it randomized a composite tissue-perfusion-guided protocol (capillary refill, skin mottling, temperature, lactate) rather than a fixed or isolated MAP target, with MAP serving only as a subordinate safety limit.

#### Data Extraction

Data were extracted independently by 2 reviewers using a standardized, prepiloted extraction form, with disagreements resolved through discussion and, when necessary, consultation with a third reviewer. Extracted variables included study characteristics (design, setting, and sample size), participant demographics and baseline clinical characteristics, relevant comorbidities (eg, chronic hypertension, chronic kidney disease, and cirrhosis), MAP intervention targets or observational exposure measures, vasopressor type and dose, and reported outcomes. Primary outcomes comprised all-cause mortality at the reported follow-up time points and splanchnic perfusion parameters. Secondary outcomes included AKI, RRT, serum creatinine, urine output, hemodynamic variables, vasopressor requirements, organ support–free days, SOFA scores, length of stay, and adverse events. Where applicable, subgroup analyses reported within individual studies were also extracted. Data presented only in graphical format were estimated from figures, and unpublished data were not requested from study investigators.

#### Risk of Bias Assessment

Methodological quality was assessed independently by 2 reviewers using validated tools according to study design, with disagreements resolved by consensus. RCTs were evaluated using the Cochrane Risk of Bias 2 (RoB 2) tool, which assesses bias arising from the randomization process, deviations from intended interventions, missing outcome data, outcome measurement, and selection of the reported results [14]. Nonrandomized studies were assessed using the Newcastle-Ottawa Scale (NOS), which evaluates study quality across the domains of selection, comparability, and outcome assessment, with a maximum score of 9 points. Overall study quality was categorized according to the respective assessment tools [15].

The remaining nonrandomized study involving 2 actively assigned MAP-targeted groups was assessed using the Risk of Bias in Non-randomized Studies of Interventions (ROBINS-I) tool, evaluating bias due to confounding, selection of participants, classification of interventions, deviations from intended interventions, missing data, outcome measurement, and selection of the reported result [16]. The single-arm, within-subject titration study lacking a comparator group was assessed using the Joanna Briggs Institute (JBI) Critical Appraisal Checklist for Quasi-Experimental Studies, a 9-item tool suited to pre-post designs without a control arm [17]. Overall quality was categorized per the respective tool applied, and studies were synthesized with attention to how these quality differences might affect confidence in the findings.

#### Data Synthesis

Given the substantial clinical and methodological heterogeneity among the included studies, the findings were synthesized narratively in accordance with the Synthesis Without Meta-Analysis reporting guideline [18]. Studies were grouped according to study design and outcome domains, with evidence from RCTs presented before nonrandomized studies. Quantitative meta-analysis was performed only for all-cause mortality because sufficient comparable interventional data were available. Separate random-effects meta-analyses were conducted for 90-day mortality and 28-day mortality, with an additional sensitivity analysis incorporating studies reporting analogous 30-day mortality. Pooled effect estimates were expressed as odds ratios (ORs) with 95% CIs, and statistical heterogeneity was assessed using Cochran *Q* test and the *I*² statistic. Meta-analysis was not undertaken for renal, hemodynamic, organ-support, or adverse event outcomes because of a lack of reported statistics, differences in study populations, MAP targets, intervention protocols, follow-up durations, and outcome definitions. Subgroup findings reported within individual studies were synthesized narratively but were not quantitatively pooled because they were primarily exploratory or post hoc analyses.

#### Certainty of Evidence

The overall certainty of the evidence for the primary outcomes was assessed using the Grading of Recommendations Assessment, Development, and Evaluation (GRADE) framework [19]. Evidence from RCTs was initially considered of high certainty and downgraded, where appropriate, for risk of bias, inconsistency, indirectness, imprecision, and publication bias. Evidence from nonrandomized studies was initially considered of low certainty and was upgraded only when justified by large effect estimates, evidence of a dose-response relationship, or when residual confounding was unlikely to explain the observed findings. Publication bias was assessed qualitatively given the small number of studies (n=3‐4) available for each pooled outcome, which precluded the reliable use of funnel plot asymmetry testing or Egger regression; this qualitative assessment considered the comprehensiveness of the search strategy, inclusion of published literature identified through multiple databases and manual reference screening, and comparison of effect directions and magnitudes across studies of differing design and setting, with no evidence suggestive of publication bias (eg, selective reporting or a disproportionate predominance of positive findings) identified. The overall certainty of evidence for each primary outcome was classified as high, moderate, low, or very low and is reported alongside the corresponding results.

## Results

### Study Selection

A systematic search was conducted across PubMed or MEDLINE, the Cochrane Library, and Google Scholar from inception through April 2026, using predefined search terms related to MAP targets, septic shock, splanchnic ischemia, and resuscitation outcomes. A total of 3254 records were identified through database searching and manual reference screening, of which 912 duplicates were removed. Following title and abstract screening, 292 full-text articles were assessed for eligibility against the prespecified inclusion criteria. Studies were excluded due to nonseptic populations, absence of a discrete MAP target comparison, insufficient outcome reporting, or nonhuman design. Ultimately, 11 studies met all inclusion criteria and were incorporated into the qualitative synthesis. The full selection process is summarized in the PRISMA flow diagram (Figure 1).

**Figure 1.**
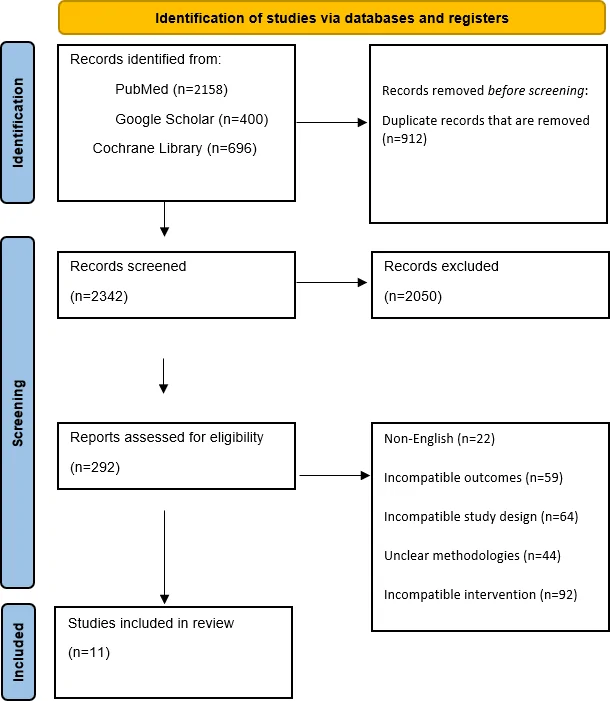
PRISMA (Preferred Reporting Items for Systematic Reviews and Meta-Analyses) flow diagram.

### Study Characteristics

#### Overview

Eleven studies (2895 patients) examined MAP targets in septic shock, including 4 RCTs and 7 nonrandomized studies (including prospective and retrospective observational cohorts, a post hoc analysis, and a physiological crossover study), with sample sizes ranging from 10 to 776. Populations were generally older adults with high illness severity (Acute Physiology and Chronic Health Evaluation II [APACHE II] 15‐29, Simplified Acute Physiology Score II [SAPS II] 38‐58, SOFA 7‐12.5), although age ranged from younger patients with cirrhosis (~45 y; Maiwall et al [20]) to an older adults-only cohort (≥65 y). Four studies compared fixed high- versus low-MAP targets (typically 80‐85 mm Hg vs 65‐70 mm Hg, except TARGET-C, which compared 80‐85 mm Hg vs 60‐65 mm Hg), whereas the remaining studies evaluated MAP as a continuous exposure or through individualized targets rather than fixed intervention arms. Comorbidities varied across cohorts, with chronic hypertension reported in 9% to 53% of patients, alongside chronic kidney disease, diabetes, coronary artery disease, and, in 1 RCT, cirrhosis with AKI (Table 1).

**Table 1.** Characteristics and results of the studies included.

Study	Design	Population (N)	Age (y)	Male (%)	Baseline severity	MAP^a^ comparison (high vs low or target)	Key comorbidities or notes
Bourgoin et al [21] (2005)	Single-center, open-label RCT^b^ (parallel-group, run-in stabilization)	Severe septic shock; N=28 (14/14)	G1: 65 (median); G2: 63 (median)	G1: 10/14 (71%); G2: 9/14 (64%)	APACHE II^c^: 27 vs 28	85 mm Hg vs 65 mm Hg (4-hour exposure)	Dopamine excluded; single vasopressor (norepinephrine) protocol
Badin et al [22] (2011)	Prospective observational cohort, 2 ICUs^d^, nonrandomized	Sustained hypotension, mixed shock etiologies; N=217 (septic shock subgroup n=127, 59%)	Mean 64 (SD 15)	127/217 (59%)	SAPS II: mean 53.2 (SD 18)	MAP as continuous exposure (no fixed arms)	Chronic HTN^e^ 41%; type 2 DM^f^ 15%; institutional MAP target >65 mm Hg
Poukkanen et al [23] (2013; FINNAKI^g^)	Prospective multicenter observational substudy, 17 ICUs, nonrandomized	Severe sepsis with continuous hemodynamic data; N=423	~63‐64 (median)	~63% overall	SAPS II^h^: 38‐43; SOFA^i^ D1: 7‐9	MAP as time-adjusted continuous exposure (no fixed arms)	HTN ~48%‐53%; CKD^j^ 11.1% vs 2.6%; DM 27.5% vs 18.5%
Endo et al [24] (2025; OPTPRESS^k^)	Multicenter, open-label RCT, Japan; stopped early for harm	Septic shock, age ≥65 y; N=516 (257/259)	High: 79 (IQR 74‐84); control: 77 (IQR 73‐85)	High: 143/257 (56%); control: 137/259 (53%)	SOFA: 9‐10; APACHE II: 25‐27	80‐85 mm Hg vs 65‐70 mm Hg	Chronic HTN ~53% (stratified variable); 97%‐99% Japanese cohort
Asfar et al [25] (2014; SEPSISPAM)^l^	Multicenter (29 centers, France), open-label RCT, stratified by chronic HTN	Septic shock refractory to fluids; N=776 (388/388)	High: mean 65 (SD 13); low: mean 65 (SD 15)	Not separately reported	SAPS II:~56‐57; SOFA:~10.7‐10.8	80‐85 mm Hg vs 65‐70 mm Hg	Chronic HTN ~44%; CKD 6%‐8%; cirrhosis ~7%
Maiwall et al [20] (2023; TARGET-C^m^)	Single-center RCT (India), block randomization	Cirrhosis + septic shock + AKI^n^; N=150 (75/75)	Low: mean 46.7 (SD 9.1); high: mean 45.0 (SD 12.9)	Low: 65/75 (87%); high: 68/75 (91%)	SOFA: ~12; MELD:~31.5‐32.5	80‐85 mm Hg vs 60‐65 mm Hg	All patients cirrhotic; HTN 9% both arms
LeDoux et al [26] (2000)	Single-center, within-patient crossover or titration study	Septic shock; N=10	Mean 68 (SD 12)	7/10 (70%)	APACHE II: 29	Sequential 65→75→85 mm Hg (no separate control arm)	Only 3/10 had prior HTN; dopamine used as co-vasoactive in 5/10 (confounder)
Dünser et al [27] (2009; Crit Care [546C88 post hoc])	Post hoc regression analysis of RCT placebo arm	Septic shock, control/placebo group; N=290 included (68 excluded, MAP <70)	Mean 60 (SD 15)	178/290 (61%)	SAPS II: 48‐50	MAP quartiles (observed exposure, not randomized)	Chronic HTN 31.4%; MV^o^ required in 96.2%
Kuragayala et al [28] (2019)	Prospective observational, single-center (India), 2 MAP groups	Septic shock refractory to fluids; N=100 (50/50)	Low: mean 58.56 (SD 18.25); high: mean 57.29 (SD 17)	Low: 28/50 (56%); high: 27/50 (54%)	SAPS II:~57‐58; SOFA:~10.4‐10.5	Target 80‐85 mm Hg vs 65‐70 mm Hg (achieved: mean 78.45 (SD 17.23) vs mean 57.32 (SD 13.69)	Chronic HTN ~19% both arms; no inferential statistics reported
Varpula et al [29] (2005)	Retrospective single-center cohort	Septic shock requiring vasopressors; N=111 (75 survivors or 36 nonsurvivors)	Survivors: mean 48.8 (SD 15.5); nonsurvivors: mean 55.3 (SD 14.6)	Survivors: 44/75 (59%); nonsurvivors: 18/36 (50%)	APACHE II: 15 vs 21; SOFA D1: 8 vs 12	MAP as continuous exposure (institutional target >65 mm Hg, no arms)	Positive blood culture ~55%; pneumonia most common source
Dünser et al [30] (2009; Bern cohort)	Retrospective single-center cohort	Sepsis or septic shock; N=274 (sepsis 126; septic shock 148)	mean 61 (SD 16)	170/274 (62%)	SOFA: mean 10.6 (SD 5.1); SAPS II: mean 51.5 (SD 19.9); APACHE II: mean 27.3 (SD 7.8)	Individualized MAP targets 50‐75 mm Hg (continuous exposure, no fixed arms)	Chronic HTN 38%; coronary heart disease 22.6%

a MAP: mean arterial pressure.

b RCT: randomized controlled trial.

c APACHE II: Acute Physiology and Chronic Health Evaluation II.

d ICU: intensive care unit.

e HTN: hypertension.

f DM: diabetes mellitus.

g FINNAKI: Finnish Acute Kidney Injury Study *(study acronym).*

h SAPS II; Simplified Acute Physiology Score II.

i SOFA: Sequential Organ Failure Assessment.

j CKD: chronic kidney disease.

k OPTPRESS: Optimal Blood Pressure in Septic Shock *(study acronym).*

l SEPSISPAM: Sepsis and Mean Arterial Pressure *(study acronym).*

m TARGET-C: Targeted Mean Arterial Pressure in Cirrhosis with Septic Shock *(study acronym).*

n AKI: acute kidney injury.

o MV: mechanical ventilation.

#### Quality Assessment

Among the 4 RCTs, 2 studies were judged to have a low overall risk of bias [24,25], whereas Bourgoin et al [21] and Maiwall et al [20] were rated as having some concerns. The main limitations were related to deviations from intended interventions, missing outcome data, and outcome measurement in the open-label trials (Figure 2).

The methodological quality of the 7 nonrandomized studies ranged from very low to good. NOS scores varied between 2 and 8, with Poukkanen et al [23] demonstrating the highest quality (8/9). Badin et al [22], Varpula et al [29], Dünser et al [27], and Dünser et al [30] achieved moderate-to-good quality (7/9; Table 2).

Of the 9 JBI items, 7 were satisfied. The 2 unmet criteria, absence of a control group (item 4) and lack of similar cotreatment across comparisons (item 3, driven by unadjusted concurrent dopamine or dobutamine use in over half the cohort), represent the principal sources of bias in this study and preclude a “low risk of bias” classification despite its otherwise rigorous, objectively measured, complete-follow-up design. The study is therefore judged to carry a moderate-to-high risk of bias, consistent with its role in this review as hypothesis-generating, physiological or mechanistic evidence rather than a source of causal effect estimates [26] (Table 3).

Kuragayala et al [28] scored 5 “Serious,” 1 “Moderate,” and 1 “Critical” domain on ROBINS-I, yielding an overall critical risk of bias due to the absence of any reported effect estimates, so the study is retained for descriptive purposes only (Figure 3).

**Figure 2.**
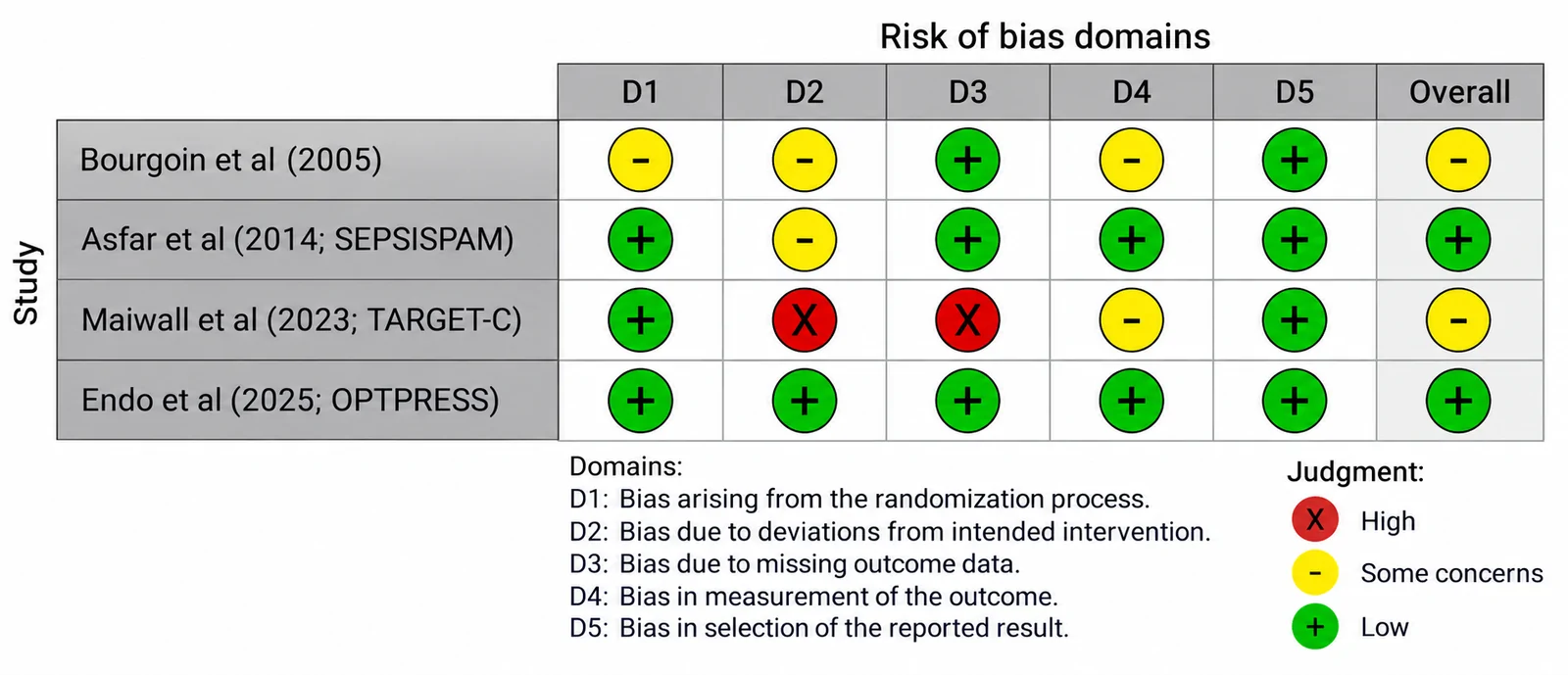
Quality assessment of randomized controlled trials (RCTs) by Risk of Bias 2 (RoB 2) tool [20,21,24,25].

**Table 2.** Newcastle-Ottawa Scale—quality assessment for observational studies.

Study	Representativeness	Selection	Exposure	Outcome absent at start	Comparability	Outcome assessment	Follow-up duration	Adequacy of follow-up	Total (/9)	Quality
Badin et al [22] (2011)	1	1	1	1	1	1	1	0	7	Moderate
Poukkanen et al [23] (2013)	1	1	1	1	2	1	1	0	8	Good
Dünser et al [27] (2009)	1	1	1	1	1	1	0	0	6	Moderate
Dünser et al [30] (2009)	1	1	1	1	1	1	1	0	7	Moderate
Varpula et al [29] (2005)	1	1	1	1	1	1	1	0	7	Good

**Table 3.** Joanna Briggs Institute (JBI) Critical Appraisal Checklist for Quasi-Experimental Studies.

Item		Judgment
1. Is it clear in the study what is the “cause” (MAP^a^ titration) and what is the “effect” (perfusion or hemodynamic response)?		Yes
2. Were the participants included in any comparisons similar?		Yes
3. Were the participants included in any comparisons receiving similar treatment or care, other than the exposure or intervention of interest?		No
4. Was there a control group?		No
5. Were there multiple measurements of the outcome, both before and after the intervention or exposure?		Yes
6. Was follow-up complete, and if not, were differences between groups in terms of their follow-up adequately described and analyzed?		Yes
7. Were the outcomes of participants included in any comparisons measured in the same way?		Yes
8. Were outcomes measured in a reliable way?		Yes
9. Was appropriate statistical analysis used?		Yes
Overall appraisal		7/9 “Yes”

a MAP: mean arterial pressure.

**Figure 3.**
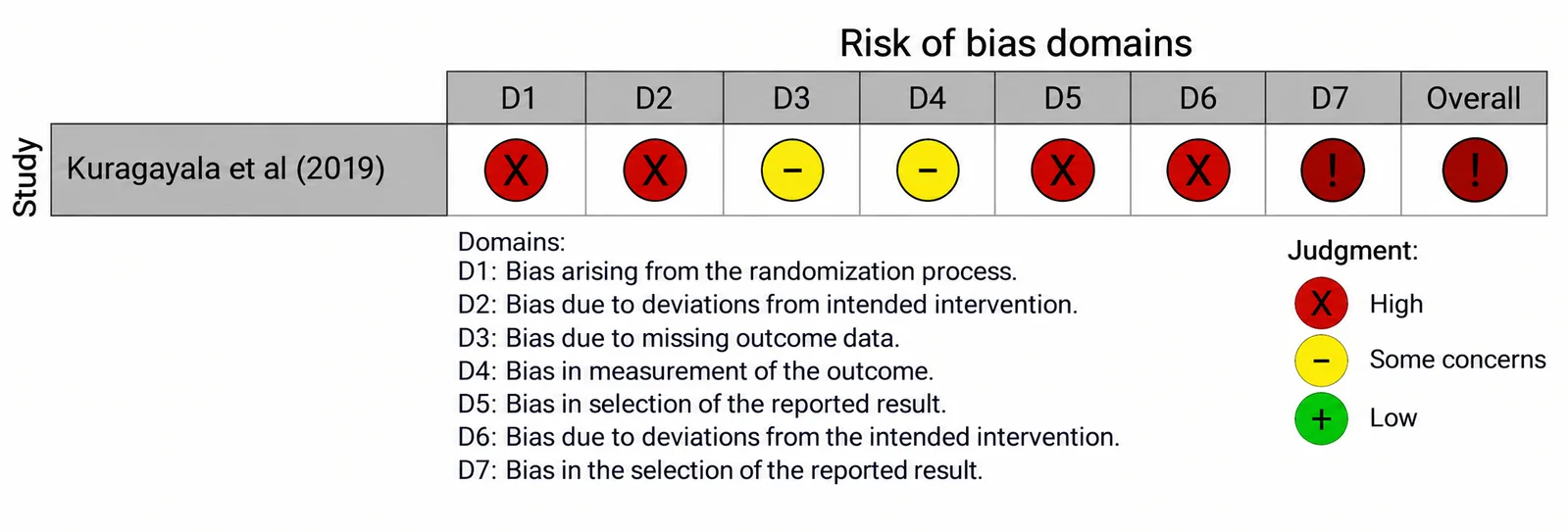
Quality assessment of nonrandomized study by Risk of Bias in Non-randomized Studies of Interventions (ROBINS I) [28].

#### GRADE

The certainty of evidence for both mortality and renal outcomes was rated as very low (Table 4). Evidence was downgraded because of methodological limitations, substantial inconsistency in the direction of treatment effects across studies, indirectness related to heterogeneous populations and MAP targets, and imprecision arising from several small studies. Consequently, the overall confidence in the effect estimates remains limited.

**Table 4.** Grading of Recommendations Assessment, Development, and Evaluation (GRADE) assessment.

Outcome	Risk of bias	Inconsistency	Indirectness	Imprecision	Publication bias	Certainty
Mortality	Serious	Very serious	Serious	Serious	Not detected	⊕◯◯◯ very low
AKI^a^ or renal outcomes	Serious	Very serious	Serious	Serious	Not detected	⊕◯◯◯ very low

a AKI: acute kidney injury.

### Primary Outcome: All-Cause Mortality

#### 90-Day Mortality

Three trials reported 90-day all-cause mortality comparing a higher versus a lower or standard MAP target and were pooled using a random-effects (Mantel-Haenszel) model [20,24,25] (Figure 4). Across 1442 patients, mortality occurred in 320 of 720 (44.4%) patients in the high-MAP arm versus 280 of 722 (38.8%) in the low-MAP arm, yielding a pooled OR of 1.31 (95% CI 0.97-1.77; *z* score=1.77; *P*=.08), a nonsignificant trend toward higher mortality with higher MAP targets. Moderate heterogeneity was present (τ²=0.03; *χ*^2^_2_=3.35, *I*^2^=40%; *P*=.19). The effect was driven mainly by Endo et al [24], whose Optimal Blood Pressure in Septic Shock (OPTPRESS) trial was stopped early for harm (OR 1.62, 95% CI 1.12-2.34) and, to a lesser extent, by Maiwall et al [20] (OR 1.48, 95% CI 0.77-2.86), while Asfar et al [25] showed an effect close to unity (OR 1.07, 95% CI 0.80-1.42; Figure 4).

**Figure 4.**
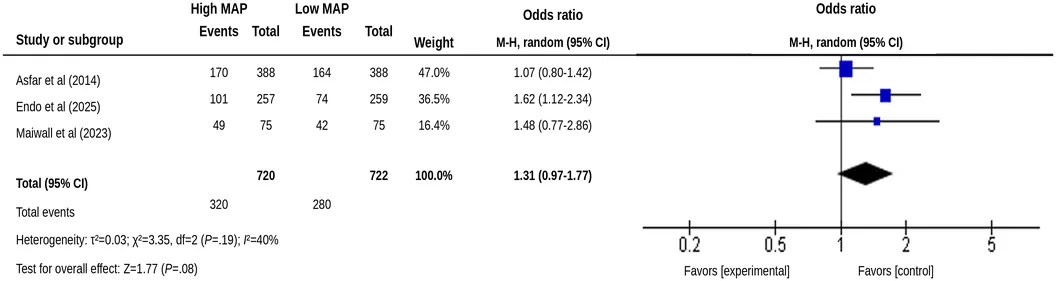
Forest plot of 90-day all-cause mortality and high- versus low-mean arterial pressure (MAP) target [20,24,25].

#### 28-Day Mortality and Sensitivity Analysis

Two trials directly reported 28-day all-cause mortality by MAP-target arm [24,25]. Because Kuragayala et al [28] reported an analogous 30-day mortality comparison, a sensitivity analysis was performed with this study both added to and removed from the pooled estimate.

With Kuragayala et al [28] included (Figure 2), pooled 28- or 30-day mortality across 3 studies (695 high-MAP, 697 low-MAP patients) was 222 of 695 (32.0%) versus 191 of 697 (27.4%), giving a pooled OR of 1.25 (95% CI 0.98-1.61; *z* score=1.78 *P*=.08), with low heterogeneity (χ^2^_2_=2.14, *I*^2^=7%; *P*=.34; Figure 5).

With Kuragayala et al [28] excluded (Figure 3), the pooled estimate from Asfar et al [25] and Endo et al [24] alone (645 patients with high-MAP, 647 patients with low-MAP) was OR of 1.31 (95% CI 0.90-1.89; *z* score=1.43; *P*=.15), with heterogeneity rising to *I*^2^ of 51% (*χ*^2^_1_=2.04; *P*=.15; Figure 6).

**Figure 5.**
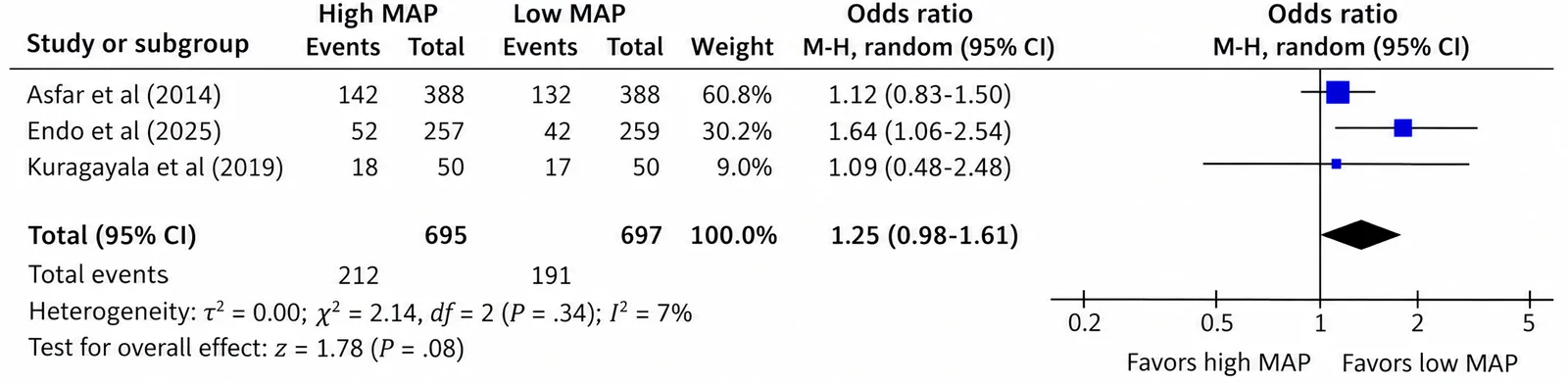
Forest plot of 28- or 30-day mortality including Kuragayala et al [28] in the sensitivity analysis. MAP: mean arterial pressure [24,25,28].

**Figure 6.**
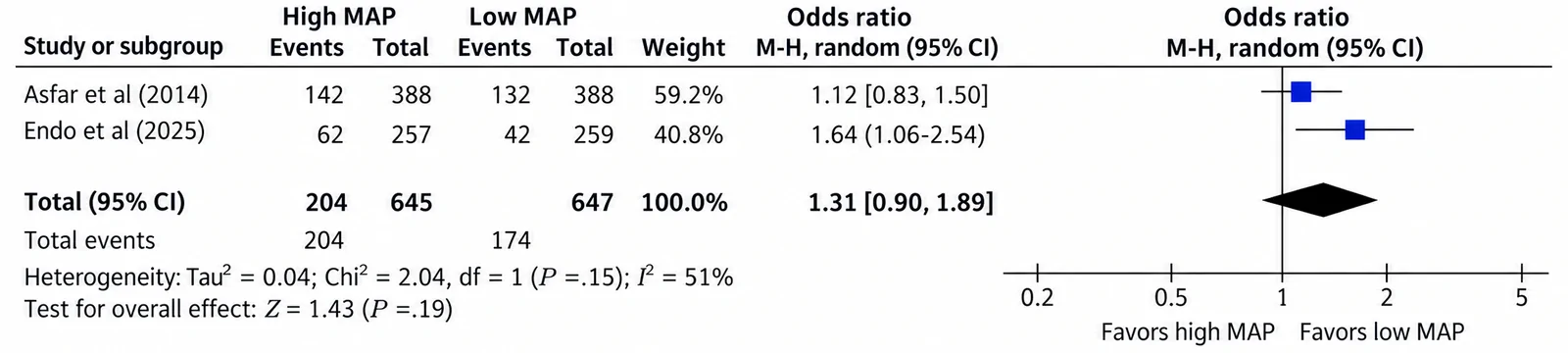
Forest plot of 28-day mortality with Kuragayala et al [28] removed in the sensitivity analysis. MAP: mean arterial pressure [24,25].

Adding Kuragayala et al [28] reduced between-study heterogeneity substantially (*I*^2^ from 51% to 7%) without materially changing the point estimate or altering statistical significance, indicating the short-term mortality result was robust to this study’s inclusion. Neither the 28- or 30-day nor the 90-day pooled estimate reached statistical significance, though both trended toward higher mortality with higher MAP targets.

#### Mortality Data Not Amenable to Pooling

Several included studies reported mortality descriptively or as a regression outcome rather than as a direct between-arm comparison and could not be pooled. Bourgoin et al [21] did not assess mortality, as the trial’s 8-hour observation window was designed only for physiologic and renal endpoints. Badin et al [22] reported ICU mortality of 35% and hospital mortality of 39% across the whole cohort without a formal statistical comparison by MAP exposure. In the Finnish Acute Kidney Injury Study (FINNAKI) substudy, Poukkanen et al [23] found that 90-day mortality was significantly higher in patients whose AKI progressed (32.7% vs 18.9%; *P*=.001), though this reflected an AKI-progression subgroup rather than a randomized MAP-target comparison. In the post hoc 546C88 cohort, Dünser et al found no significant association between MAP quartile and 28-day mortality after adjustment (risk ratio [RR] 0.99, 95% CI 0.95-1.04, *P*=.82), although patients excluded for failing to sustain MAP ≥70 mm Hg had markedly higher 28-day mortality (76.5%) than the included cohort (42.1%). Conversely, Dünser et al [27] found that MAP was among the strongest independent predictors of 28-day mortality in both 6-hour (*P*=.001) and 48-hour (*P*=.01) logistic regression models, with the largest area under the receiver operating characteristic curve (AUC) for time spent below MAP 60 mm Hg (AUC=0.779) and no significant difference in discriminative performance between MAP thresholds of 60 and 75 mm Hg [30]. Similarly, Varpula et al [29] found that each 1 mm Hg decrease in mean 48-hour MAP was independently associated with an approximate 16% increase in the odds of death (Exp[B]=1.156; *P*=.01).

### Secondary Outcomes

#### AKI and RRT

Renal outcomes were reported with heterogeneous definitions across 7 studies, precluding meta-analytic pooling. Bourgoin et al found that acutely raising MAP from 65 to 85 mm Hg over 4 hours did not change urine flow, serum creatinine, or creatinine clearance [21]. Badin et al [22] identified an MAP range of 72 to 82 mm Hg as the best discriminator of AKI progression at 72 hours, but only in patients with septic shock with early (6-h) renal impairment (AUC=0.83‐0.84); no MAP-AKI association was found in patients without early AKI. In the larger FINNAKI substudy, Poukkanen et al [23] reported that a time-adjusted MAP below 73 mm Hg was independently associated with AKI progression after adjustment for chronic kidney disease, lactate, furosemide dose, and dobutamine use (adjusted OR 2.57, 95% CI 1.48-4.46). In Sepsis and Mean Arterial Pressure (SEPSISPAM), Asfar et al [25] found no overall between-group difference in creatinine doubling or RRT, but among patients with chronic hypertension, the high-MAP group had significantly less RRT use (31.7% vs 42.2%; *P*=.046) and less creatinine doubling (38.9% vs 52.0%; *P*=.02). In patients with cirrhosis with septic shock and AKI, Maiwall et al [20] found AKI reversal at day 5 was significantly greater with the high-MAP target in the per-protocol analysis (53% vs 31%; *P*=.02), and a high-MAP strategy independently predicted AKI recovery (OR 2.36‐2.47). Kuragayala et al [28] reported similar, purely descriptive rates of creatinine doubling (82% vs 84%) and RRT (66% vs 70%) between arms, without inferential statistics. Finally, Dünser et al [30] found that MAP and mean perfusion pressure were both significantly correlated with RRT need (r^2^=0.124‐0.136; *P*≤.001) and peak creatinine (*r*^2^=0.269‐0.277), with MAP <75 mm Hg providing the best discrimination for subsequent RRT need (AUC=0.724).

#### Hemodynamic Parameters and Vasopressor Requirements

A consistent, dose-dependent relationship between MAP target and vasopressor exposure was observed across studies. In a within-patient crossover from 65 to 75 mm Hg to 85 mm Hg, LeDoux et al [26] demonstrated a stepwise linear increase in heart rate, cardiac index, left ventricular stroke work index, systemic vascular resistance index, and norepinephrine dose (from 23 to 31 µg/min to 47 µg/min; *P*=.02). Bourgoin et al similarly found that raising MAP from 65 to 85 mm Hg significantly increased cardiac index and systemic vascular resistance index without changing lactate or oxygen consumption [21]. Among the RCTs, higher MAP targets consistently required substantially greater vasopressor exposure: Asfar et al [25] reported higher day-1 norepinephrine dose (0.58 vs 0.45 µg/kg/min; *P*<.001) and longer catecholamine infusion (4.7 vs 3.7 days; *P*<.001) in the high-MAP arm, Endo et al [24] reported longer vasopressor duration (65 vs 42 h) and higher cumulative norepinephrine (15.6 vs 9.6 mg) and vasopressin doses in the high-target arm, and Kuragayala et al [28] noted the low-MAP arm undershot its 65- to 70-mm Hg target (achieved MAP 57.3±13.7 mm Hg), while the high-MAP arm approximated its 80- to 85-mm Hg target (78.5±17.2 mm Hg).

#### Organ Support, Mechanical Ventilation, and Length of Stay

Endo et al [24] reported significantly fewer RRT-free days (18 vs 20; *P*=.02), ventilator-free days (15 vs 18; *P*=.012), and catecholamine-free days (16 vs 19; *P*=.01) at 28 days with the high-target strategy, indicating a greater overall organ-support burden. In contrast, Asfar et al [25] found no significant difference in 28-day survival without organ support between arms (60.6% vs 62.1%; *P*=.66), and Maiwall et al [20] found no significant intention-to-treat differences in ICU length of stay or mechanical ventilation duration, although both trended longer in the high-MAP arm. In the FINNAKI substudy, patients with AKI progression had significantly longer ICU stays than those without (5.7 vs 3.8 d; *P*<.001) [23].

#### Adverse Events and Splanchnic or Ischemic Outcomes

None of the included studies directly reported splanchnic perfusion or ischemia as a discrete primary outcome; the closest available proxy was mesenteric ischemia as an adverse event, which occurred at similar, low rates between arms in both Asfar et al [25] (2.3% vs 2.3%) and Maiwall et al [20] (1.3% high-MAP vs 0% low-MAP). Cardiac arrhythmias were more frequent with higher MAP targets in several studies: Asfar et al [25] reported significantly more atrial fibrillation with high MAP (6.7% vs 2.8%, *P*=.02), and Endo et al [24] noted that all main safety outcomes—arrhythmia, ischemic events, and hemorrhagic events—trended more frequent in the high-target group, although none reached statistical significance individually. Maiwall et al [20] found significantly more adverse events requiring protocol discontinuation with high MAP (24% vs 11%; *P*=.03), but significantly less intradialytic hypotension (12% vs 48%; *P*<.001), suggesting a trade-off between systemic vasopressor-related harm and protection from dialysis-associated hypotension at higher MAP targets.

## Discussion

### Principal Findings

This systematic review synthesized evidence from 11 studies examining MAP targets during septic shock resuscitation, with particular attention to splanchnic perfusion and renal outcomes. Pooled analysis of 3 trials [20,24,25] showed a nonsignificant trend toward higher 90-day mortality with higher MAP targets (OR 1.31, 95% CI 0.97-1.77), a trend echoed in the pooled 28- or 30-day mortality estimate derived from Asfar et al [25], Endo et al [24], and, in sensitivity analysis, Kuragayala et al [28] (OR 1.25, 95% CI 0.98-1.61). Although neither estimate reached statistical significance, the consistency of direction across both time points, combined with the early termination of the OPTPRESS trial due to harm [24], raises genuine concern that targeting a higher MAP does not confer a survival benefit and may plausibly increase risk in some patients.

These findings diverge from the physiological rationale that historically supported higher MAP targets in septic shock. Early mechanistic work by LeDoux et al [26] and Bourgoin et al [21] showed that acutely raising MAP from 65 to 85 mm Hg increased cardiac index and systemic vascular resistance without improving lactate clearance, urine flow, or creatinine clearance, suggesting that macrocirculatory augmentation above 65 mm Hg does not reliably translate into organ-level perfusion benefit. This disconnect persisted into the RCT era: Asfar et al [25] found no overall mortality or organ support–free survival benefit with an 80‐ to 85-mm Hg target, and Endo et al [11] found the higher target reduced RRT-free, ventilator-free, and catecholamine-free days at 28 days.

A recurring theme across the evidence base, however, is that the relationship between MAP and organ injury is not uniform across patients and that baseline physiology modifies both the direction and magnitude of treatment effect. The clearest example is chronic hypertension: within SEPSISPAM, Asfar et al [25] reported that patients with hypertension randomized to the higher MAP target had significantly less RRT use (31.7% vs 42.2%; *P*=.046) and less creatinine doubling (38.9% vs 52.0%; *P*=.02), an effect not observed in patients with normal blood pressure. This is consistent with a rightward-shifted renal autoregulatory curve in patients with chronic hypertension, whereby an MAP adequate for autoregulation in normotensive individuals may fall below the autoregulatory threshold in those with long-standing hypertension. A second example is cirrhosis: Maiwall et al [20] found that a higher MAP target improved AKI reversal at day 5 in patients with cirrhosis and septic shock and AKI (53% vs 31%; *P*=.02), a population characterized by splanchnic vasodilation, effective arterial underfilling, and impaired renal autoregulation distinct from the general septic shock population. These subgroup signals, alongside the broader trial-level null or harm findings, suggest that a single population-wide MAP target may obscure clinically important heterogeneity of treatment effect and that individualized, comorbidity-informed targets may be more physiologically appropriate than fixed thresholds.

The renal outcome data reinforce this picture of MAP as a threshold rather than a linear “higher is better” variable. Poukkanen et al [23] identified a time-adjusted MAP below 73 mm Hg as independently associated with AKI progression (adjusted OR 2.57, 95% CI 1.48-4.46), while Dünser et al [30] reported that time below an MAP of 60 mm Hg was the strongest discriminator of 28-day mortality (AUC=0.779), with no added benefit from thresholds between 60 and 75 mm Hg. Similarly, Badin et al [22] found that an MAP of 72‐82 mm Hg best discriminated AKI progression only in patients with existing early renal impairment. Collectively, these data suggest a threshold effect in which avoiding hypotension below roughly 60 to 65 mm Hg is critical, but pushing MAP higher confers diminishing or absent renal benefit, consistent with the null or harmful mortality signals from Asfar et al [25] and Endo et al [24].

Notably, none of the included studies reported splanchnic perfusion or ischemia as a discrete primary outcome, despite this being a central rationale for the review. The only available proxy, mesenteric ischemia as an adverse event, occurred at low and similar rates between MAP arms in both Asfar et al [25] (2.3% vs 2.3%) and Maiwall et al [20] (1.3% vs 0%), providing some reassurance that higher MAP targets are not associated with excess overt bowel ischemia, but this outcome was underpowered and nonspecific and cannot be equated with subclinical splanchnic hypoperfusion [20,25]. This represents an important evidence gap rather than evidence of absence of effect.

The safety profile of higher MAP targets also warrants attention. Higher targets were consistently associated with greater vasopressor exposure, from the stepwise norepinephrine increases seen by LeDoux et al to the significantly higher day-1 and cumulative catecholamine doses reported by Asfar et al [25] and Endo et al [24,26]. This increased vasopressor burden was accompanied by a higher incidence of atrial fibrillation in Asfar et al [25] (6.7% vs 2.8%; *P*=.02) and a trend toward more arrhythmic, ischemic, and hemorrhagic events in Endo et al [24]. Maiwall et al [20] identified a clinically important trade-off in patients with cirrhosis, in whom higher MAP targets increased protocol-discontinuing adverse events (24% vs 11%; *P*=.03) but substantially reduced intradialytic hypotension (12% vs 48%; *P*<.001), illustrating that the net safety balance of a given MAP target may depend heavily on which complications are most relevant to a specific population.

Our findings both extend and refine the conclusions of prior reviews. Leone et al [8], synthesizing studies through July 2014, concluded that an MAP of 65 mm Hg was generally sufficient, although 75 to 85 mm Hg might reduce AKI in patients with chronic hypertension. Our review, incorporating a decade of additional evidence including SEPSISPAM, Targeted Mean Arterial Pressure in Cirrhosis with Septic Shock (TARGET-C), and OPTPRESS, corroborates this hypertension-specific renal signal but adds a caveat Leone et al [8] could not have anticipated: the subsequent OPTPRESS trial [24], conducted in older patients, was stopped early for harm, indicating that higher-MAP benefit in hypertensive or older populations is not generalizable and may reverse in advanced age. Dari et al [10], restricting their meta-analysis to 4 RCTs, similarly found no significant difference in mortality (RR 0.94, 95% CI 0.87-1.01), RRT use, or ICU stay between high- and low-normal MAP targets. However, by excluding observational evidence entirely, Dari et al [10] could not capture the continuous, threshold-based MAP–outcome relationship shown in cohorts such as Poukkanen et al [23], Varpula et al [29], and Dünser et al [27,30], nor the cirrhosis-specific findings of Maiwall et al [20] that our broader inclusion criteria captured. Kattan et al [11], reviewing hemodynamic monitoring, argued for shifting away from fixed macrohemodynamic targets such as MAP toward multimodal, perfusion-based endpoints such as capillary refill time. Our findings lend indirect support: the absence of consistent mortality or renal benefit from higher MAP across heterogeneous populations, alongside dose-dependent escalation in vasopressor exposure and arrhythmic risk, suggests that a single macrohemodynamic number is an imprecise proxy for tissue perfusion, reinforcing the case for perfusion-guided, individualized resuscitation over uniform MAP thresholds.

### Limitations

Several limitations should be considered. First, substantial clinical and methodological heterogeneity across included studies, spanning populations (general septic shock, cirrhosis, older adults-only cohorts), MAP comparison strategies (fixed targets vs continuous exposure), and outcome definitions, precluded meta-analysis for all outcomes except mortality. Second, the overall certainty of evidence for mortality and renal outcomes was rated as very low using GRADE, reflecting serious risk of bias, inconsistency, and imprecision. Third, several trials were open-label, and 2 RCTs [20,21] were rated as having some concerns on RoB 2. Fourth, this review was not prospectively registered on PROSPERO, and restriction to English-language, full-text-available studies may have introduced language bias. Fifth, reliance on Google Scholar screening limited to the first 400 relevance-ranked results may have missed additional eligible studies. Sixth, this review itself included only 4 RCTs, a limitation similar to those it critiques in prior reviews. Seventh, adverse events were systematically reported in only 3 of the included studies, and arrhythmia data derive from just 2 trials (Asfar et al [25], Endo et al [24]), limiting the generalizability of the safety findings. Finally, no included study directly measured splanchnic perfusion or ischemia, forcing reliance on an imprecise adverse-event proxy that cannot capture subclinical gut hypoperfusion.

### Future Directions

Future research should prioritize direct, quantitative measurement of splanchnic and mesenteric perfusion, using gastric tonometry, sublingual microcirculatory imaging, near-infrared spectroscopy, or gut-barrier biomarkers, within randomized MAP-target comparisons. Given the signal of differential treatment effect by comorbidity, adequately powered trials or individual patient data meta-analyses stratified by chronic hypertension, cirrhosis, and age are needed to clarify which subgroups benefit from higher MAP targets, and to reconcile the hypertension-related benefit reported by Leone et al [8] and Asfar et al [25] with the harm signal in older patients seen in Endo et al [24]. Trials should incorporate standardized vasopressor and adverse-event reporting to enable pooled safety analyses and should consider individualized or perfusion-guided targets, as advocated by Kattan et al [11], rather than fixed population thresholds. Finally, prospective registration and consistent outcome definitions across future studies would improve the poolability of evidence in this field.

### Conclusion

Overall, this review does not support routinely targeting a higher MAP in septic shock, as the available evidence points toward no meaningful survival benefit and, if anything, a possible increase in harm, alongside greater vasopressor exposure and cardiovascular risk. At the same time, the evidence suggests that MAP is not a truly one-size-fits-all target: certain patients, particularly those with chronic hypertension or underlying liver disease, may benefit from a somewhat higher pressure target to protect kidney function, reflecting differences in how these patients’ bodies regulate blood flow. Importantly, none of the available studies directly examined gut perfusion, leaving the central question motivating this review unanswered. Given the overall low certainty of the current evidence, a standard MAP target remains the safest general approach for most patients with septic shock, while an individualized, slightly higher target may be reasonable in select high-risk subgroups. Future research should move beyond a single blood pressure number and instead focus on directly assessing tissue perfusion, tailoring resuscitation strategies to the individual patient rather than applying a uniform target to all.

## Supplementary material

10.2196/98772 Checklist 1 PRISMA checklist.

## Abbreviations

AKI: acute kidney injury
APACHE II: Acute Physiology and Chronic Health Evaluation II
AUC: area under the receiver operating characteristic curve
CENTRAL: Cochrane Central Register of Controlled Trials
FINNAKI: Finnish Acute Kidney Injury Study
GRADE: Grading of Recommendations Assessment, Development, and Evaluation
ICU: intensive care unit
JBI: Joanna Briggs Institute
MAP: mean arterial pressure
NOS: Newcastle-Ottawa Scale
OPTPRESS: Optimal Blood Pressure in Septic Shock
OR: odds ratio
PICO: Population, Intervention, Comparator, and Outcome
PRISMA: Preferred Reporting Items for Systematic Reviews and Meta-Analyses
RCT: randomized controlled trial
RoB 2: Risk of Bias 2
ROBINS-I: Risk of Bias in Non-randomized Studies of Interventions
RR: risk ratio
RRT: renal replacement therapy
SAPS II: Simplified Acute Physiology Score II
SEPSISPAM: Sepsis and Mean Arterial Pressure
SOFA: Sequential Organ Failure Assessment
TARGET-C: Targeted Mean Arterial Pressure in Cirrhosis with Septic Shock
